# Exploring the mechanism of action of dapansutrile in the treatment of gouty arthritis based on molecular docking and molecular dynamics

**DOI:** 10.3389/fphys.2022.990469

**Published:** 2022-08-29

**Authors:** Jun-Feng Cao, Xingyu Yang, Li Xiong, Mei Wu, Shengyan Chen, Hengxiang Xu, Yunli Gong, Lixin Zhang, Qilan Zhang, Xiao Zhang

**Affiliations:** ^1^ Clinical Medicine, Chengdu Medical College, Chengdu, China; ^2^ Laboratory Medicine, Chengdu Medical College, Chengdu, China; ^3^ Yunnan Academy of Forestry Sciences, Kunming, Yunnan, China; ^4^ Chengdu Medical College of Basic Medical Sciences, Chengdu, China

**Keywords:** gouty arthritis, dapansutrile, NLRP3, molecular docking, molecular dynamics

## Abstract

**Purpose:** Dapansutrile is an orally active β-sulfonyl nitrile compound that selectively inhibits the NLRP3 inflammasome. Clinical studies have shown that dapansutrile is active *in vivo* and limits the severity of endotoxin-induced inflammation and joint arthritis. However, there is currently a lack of more in-depth research on the effect of dapansutrile on protein targets such as NLRP3 in gouty arthritis. Therefore, we used molecular docking and molecular dynamics to explore the mechanism of dapansutrile on NLRP3 and other related protein targets.

**Methods:** We use bioinformatics to screen active pharmaceutical ingredients and potential disease targets. The disease-core gene target-drug network was established and molecular docking was used for verification. Molecular dynamics simulations were utilized to verify and analyze the binding stability of small molecule drugs to target proteins. The supercomputer platform was used to measure and analyze the binding free energy, the number of hydrogen bonds, the stability of the protein target at the residue level, the radius of gyration and the solvent accessible surface area.

**Results:** The protein interaction network screened out the core protein targets (such as: NLRP3, TNF, IL1B) of gouty arthritis. Gene ontology (GO) and Kyoto Encyclopedia of Genes and Genomes (KEGG) analysis revealed that gouty arthritis mainly played a vital role by the signaling pathways of inflammation and immune response. Molecular docking showed that dapansutrile play a role in treating gouty arthritis by acting on the related protein targets such as NLRP3, IL1B, IL6, etc. Molecular dynamics was used to prove and analyze the binding stability of active ingredients and protein targets, the simulation results found that dapansutrile forms a very stable complex with IL1B.

**Conclusion:** We used bioinformatics analysis and computer simulation system to comprehensively explore the mechanism of dapansutrile acting on NLRP3 and other protein targets in gouty arthritis. This study found that dapansutrile may not only directly inhibit NLRP3 to reduce the inflammatory response and pyroptosis, but also hinder the chemotaxis and activation of inflammatory cells by regulating IL1B, IL6, IL17A, IL18, MMP3, CXCL8, and TNF. Therefore, dapansutrile treats gouty arthritis by attenuating inflammatory response, inflammatory cell chemotaxis and extracellular matrix degradation by acting on multiple targets.

## Graphical Abstract

The mechanisms analysis of dapansutrile in the treatment of gouty arthritis.
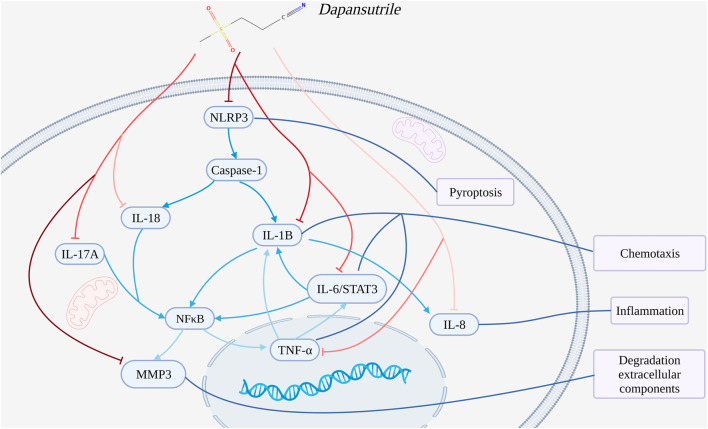

## Introduction

Gouty arthritis is considered one of the most common forms of inflammatory arthritis, it is a metabolic rheumatic disease (Roddy and Choi, 2014; Kim et al., 2016; Xie et al., 2017). Gouty arthritis is a form of arthritis caused by deposits of uric acid crystals called monosodium urate (MSU) crystals. The disease is usually secondary to chronic hyperuricemia, and the lesions are located in the joints and bursae (Klück et al., 2020; Wang and Wang, 2020). MSU crystal-induced gouty arthritis can occur in joints, periarticular tissues and kidneys (Wu et al., 2015). Acute gouty arthritis (AGA) is usually characterized by joint redness, swelling, warmth, and pain. As the disease progresses, gouty arthritis eventually leads to deformity of the diseased joint and severe limitation of joint movement (Yu et al., 2022). The principal clinical treatment goals for gouty arthritis are stopping acute attacks, preventing recurrences and complications. The American College of Rheumatology (ACR) guidelines for the treatment of gout published in 2012 recommend the use of non-steroidal anti-inflammatory drugs (NSAIDs) or oral colchicine for the treatment of acute gout attacks (Khanna et al., 2012). It has been reported that NSAIDs are harmful to the gastrointestinal tract, liver and kidney, central nervous system, etc. (Bindu et al., 2020). Colchicine is an anti-inflammatory drug, which is widely used to treat acute gouty arthritis. However, colchicine can cause gastrointestinal discomfort, liver and kidney damage and multi-organ dysfunction when taken in high doses (Blackham et al., 2007). At the same time, it is under a narrow therapeutic index with no significant difference between non-toxic, toxic and lethal doses, which may lead to toxicity in patients (Finkelstein et al., 2010). Therefore, there is an imperative for a safe and effective drug for the treatment of gouty arthritis.

Dapansutrile (OLT1177) is a beta-sulfonitrile compound. It can inhibit the NLRP3 inflammasome and reverses the metabolic cost of inflammation (Marchetti et al., 2018a). The drug was originally formulated as a candidate for the topical treatment of degenerative arthritis, and an oral form was subsequently developed. As with topical gels, oral capsules have been shown to be safe and well tolerated in humans (Marchetti et al., 2018a; Marchetti et al., 2018b). Dapansutrile has been shown to be safe in humans, and Dapansutrile was the first NLRP3 inhibitor to complete two human proof-of-concept studies, one for acute gouty arthritis flares (Phase 2a) and one for stable systolic heart failure (NYHA II-III) (Phase 1b) (Aliaga et al., 2021). Dapansutrile has been shown to specifically block NLRP3 inflammasome formation and prevent caspase-1 activation and IL1B maturation and release (Yang et al., 2019; Lonnemann et al., 2020). NLRP3 inflammasome formation also induces pyroptosis (Jorgensen and Miao, 2015). Interestingly, dapansutrile reduced neutrophil infiltration and joint swelling and inhibited the secretion of pro-inflammatory factors IL1B, IL18, and IL6 in a mouse model of yeast glycan- and urate-induced arthritis (Marchetti et al., 2018b; Zhao and Zhao, 2020). At the same time, activation of the NLRP3 inflammasome induces the maturation of IL1B and IL18, both of which are effective targets for the treatment of acute and chronic inflammatory diseases (Dinarello et al., 2012).

However, there is currently a lack of more in-depth and systematic research on dapansutrile in the treatment of gouty arthritis. Molecular dynamics can comprehensively and systematically simulate the interaction and binding stability between small molecule monomers and protein targets with the help of powerful computing power.

Molecular dynamics (MD) use large computer clusters as the carrier to obtain data such as the microstructure, physicochemical properties, and performance characterization parameters of small molecule drugs and proteins through calculation (Santos et al., 2019). It is a supplement and in-depth exploration of traditional biomedical disciplines based on experiments. Through the data obtained by calculation, the mechanism behind the experiment is analyzed from the micro, meso and macro scales of multi-level research. Molecular dynamics analyzes the behavioral laws of molecular motion by solving the potential functions and motion equations of intermolecular interactions, it simulates the dynamic evolution process of the system, and it provides microscopic quantities (such as the coordinates and velocity of molecules) and macroscopic observable quantities (such as: the relationship between the temperature, pressure, heat capacity of the system, etc.) (Sivakumar et al., 2020), so as to study the equilibrium properties and mechanical properties of composite systems. Therefore, molecular dynamics can be systematically and comprehensively analyze the stability and affinity of dapansutrile and gouty arthritis related protein targets.

Since the mechanism of action of dapansutrile in the treatment of gouty arthritis is still unclear, this study used bioinformatics to screen core targets between dapansutrile and gouty arthritis, and we used gene ontology (GO), Kyoto Encyclopedia of Genes and Genomes (KEGG) and protein-protein interaction (PPI) to analyze target genes and explore their mechanisms of action and potential pathways. We used molecular system motions to simulate the results of computing interactions from the cellular level to the chemical group level. Molecular docking was used to determine the affinity of monomeric compounds to protein targets, and molecular dynamics were used to model the stability of bound complexes. Research on the mechanism of action of dapansulide in the treatment of gouty arthritis will promote the related research and clinical application of the drug.

## Materials and methods

### Core gene targets screening and protein-protein interaction network building

“Gouty arthritis” was used to be the key word to obtain the disease gene targets through GeneCards database. The STRING database was used to analyze the protein-protein interaction (PPI) of dapansutrile in the treatment of gouty arthritis (Szklarczyk et al., 2019). In order to further clarify the interaction between potential protein targets, all potential therapeutic protein targets of dapansutrile on gouty arthritis were imported into Cytoscape 3.7.1 to analyze (Shannon et al., 2003), we defined the protein type as “*Homo sapiens*,” and obtained relevant information on protein interactions by STRING database. Finally, the network topology parameters were analyzed by Cytoscape 3.7.1, and the core protein targets were screened out according to the criterion that the node degree value and the betweenness center value were greater than the average value.

### The gene target enrichment analysis

The main biological processes and signaling pathways of dapansutrile on gouty arthritis were analyzed though DAVID database. The interaction gene targets were used in DAVID database for gene ontology (GO) functional annotation and Kyoto Encyclopedia of Genes and Genomes (KEGG) enrichment analysis. We obtained molecular function (MF), cellular component (CC) and biological process (BP) of the gene targets through GO enrichment. The disease related targets obtained from screening were input into the DAVID database by entering the list of target gene names and selecting the species as “*Homo sapiens*” (Huang et al., 2009). In this study, KEGG pathway enrichment analysis was performed on the relevant signaling pathways involved in the target, and gene target screening was performed under the condition of *p* < 0.05 (Cao et al., 2022).

### Network diagram of “disease-core target gene-drug”

This study used the Cytoscape 3.7.1 network map software to construct a disease-core target gene-drug network and conduct topological analysis. The core gene targets can be screened based on the node degree value greater than 2 times the median (Cao et al., 2022).

### Molecular docking and validation of the docking protocol

Molecular docking was used to study the molecular affinity of dapansutrile with gouty arthritis protein targets. In this study, AutoDock Vina 1.1.2 software was used for molecular docking work. Before docking, PyMol 2.5 was used to process all receptor proteins, including removal of water molecules, salt ions and small molecules (Kim et al., 2016). ADFRsuite 1.0 was used to convert all processed small molecules and receptor proteins into the PDBQT format necessary for docking with AutoDock Vina 1.1.2. The output highest scoring docked conformation was considered to be the binding conformation for subsequent molecular dynamics simulations (Kim et al., 2016). The study used the original crystal ligand of the protein target as a positive reference, and we analyzed and compared the binding posture of the original crystal ligand and protein, the chemical bond length and the chemical bond angle by re-docking the original crystal ligand and protein. Finally, the consistency of the binding mode could indicate the correctness of the molecular docking protocol (Cao et al., 2022).

### Molecule dynamics

In this study, the stability of the conformational complex of the dapansutrile and gouty arthritis proteins was further verified by molecular dynamics simulations. Molecular dynamics (MD) simulation is a comprehensive set of molecular simulation methods combining physics, mathematics and chemistry. This method mainly relies on Newtonian mechanics to simulate the motion of molecular systems, we calculate macroscopic properties such as thermodynamic quantities of a system by taking samples from an ensemble of different states of a molecular system (Burley et al., 2017). In this study, the small molecules and protein complexes obtained from the molecular docking results were used as the initial structures, and AMBER 18 software was used to conduct all-atom molecular dynamics simulations (Maier et al., 2015). The charge of the small molecule was calculated in advance by the antechamber module and the Hartree-Fock (HF) SCF/6-31G* of the gaussian 09 software before the simulation (Harrach and Drossel, 2014). Finally, the simulated topology and parameter files were exported. After the initial addition of hydrogen atoms to each system, the system used a steepest descent algorithm for vacuum minimization (Wang et al., 2006). The solvent was then added and the system ions were balanced with counter ions (Na+/Cl−).

The proteins were all energy minimized using the steepest descent method and the conjugate gradient method. Subsequently, combined NVT and NPT (1,000 ps, 2 fs dt) and MD tests (100 ns, 2 fs dt) were performed at 298 K temperature and 1 bar pressure using a jump-integration algorithm. The coordinates and energy of the system are saved every 10 ps. Finally, 50 ns production simulations were carried out for each system under periodic boundary conditions. For all simulations, the van der Waals force (vdw) cutoff and short-range electrostatic interactions were set to 10 Å. The Particle-Mesh-Ewald (PME) method was used to evaluate long-range electrostatic interactions. Molecular dynamics simulation trajectories include protein-ligand complex root mean square deviation (RMSD), root mean square fluctuation (RMSF), radius of gyration and solvent accessible surface area (SASA).

### MMGBSA binding free energy calculation

In this study, the binding free energy of the compound was investigated by MM-PBSA method, and its conformational stability was studied in detail. We calculated the binding free energies between proteins and ligands in all systems using the MM/GBSA method (Hou et al., 2011). The molecular dynamics trajectory of 50 ns was used for calculation, and the specific formula was as follows:

$$\Delta G_{\text{bind}}=\Delta G_{\text{complex}}-\left(\Delta G_{\text{receptor}}+\Delta G_{\text{ligand}}\right)=\Delta {Ε}_{\text{internal}}+\Delta {Ε}_{\text{VDW}}+\Delta {Ε}_{\text{elec}}+\Delta GL_{\text{GB}}+\Delta G_{\text{SA}}$$

In the formula, bond energy (*E*_bond_), angular energy (*E*_angle_), torsion energy (*E*_torsion_), *ΔG*_GA_ and *ΔG*_GB_ are collectively called solvation free energy. The non-polar solvation free energy (*ΔG*_GA_) was calculated based on the product of surface tension (*γ*) and solvent accessible surface area (SA), *ΔG*_GA_ = 0.0072 × SASA (Cao et al., 2022).

## Results

### Core target screening and protein-protein interaction network diagram

**FIGURE 1:**
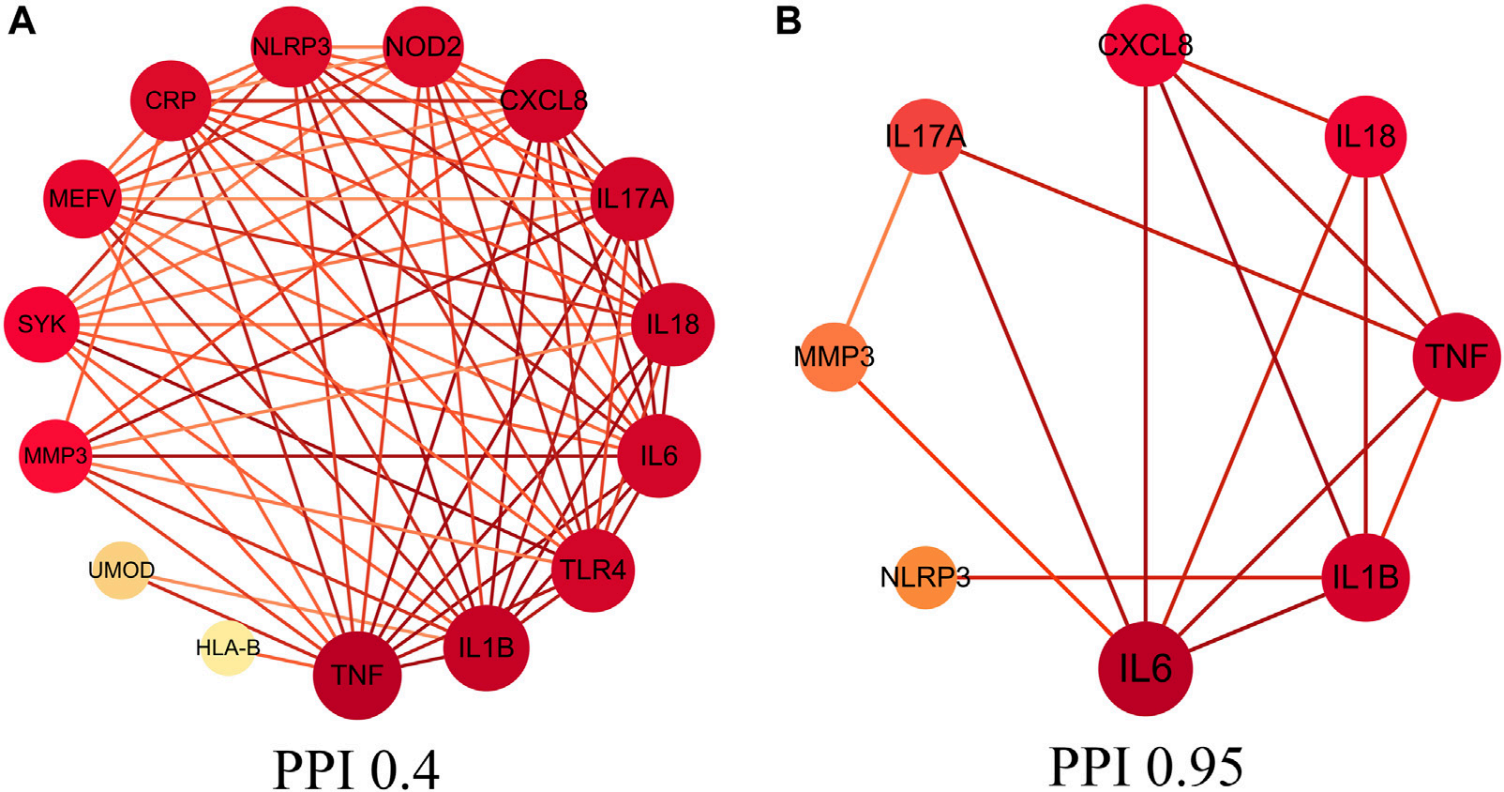
Protein-protein interaction (PPI) network. **(A)** PPI network of protein target, **(B)** PPI network of core protein target (confidence > 0.95).

A total of 220 gouty arthritis gene targets was screened though GeneCards database. We obtained core genes targets though relevance score, relevance score ≥20 which was considered as core gene target, the study analyzed 18 core gene targets through STRING database to construct the PPI network interaction map of target proteins of dapansutra for gouty arthritis, shown in Figure 1A. Eight core genes (such as: NLRP3, IL1B, CXCL8, etc.) were obtained by improving the confidence score (confidence degree > 0.95), and the study used the eight core gene targets to construct the core PPI network, shown in Figure 1B.

### Gene ontology and Kyoto Encyclopedia of Genes and Genomes enrichment analysis

**FIGURE 2:**
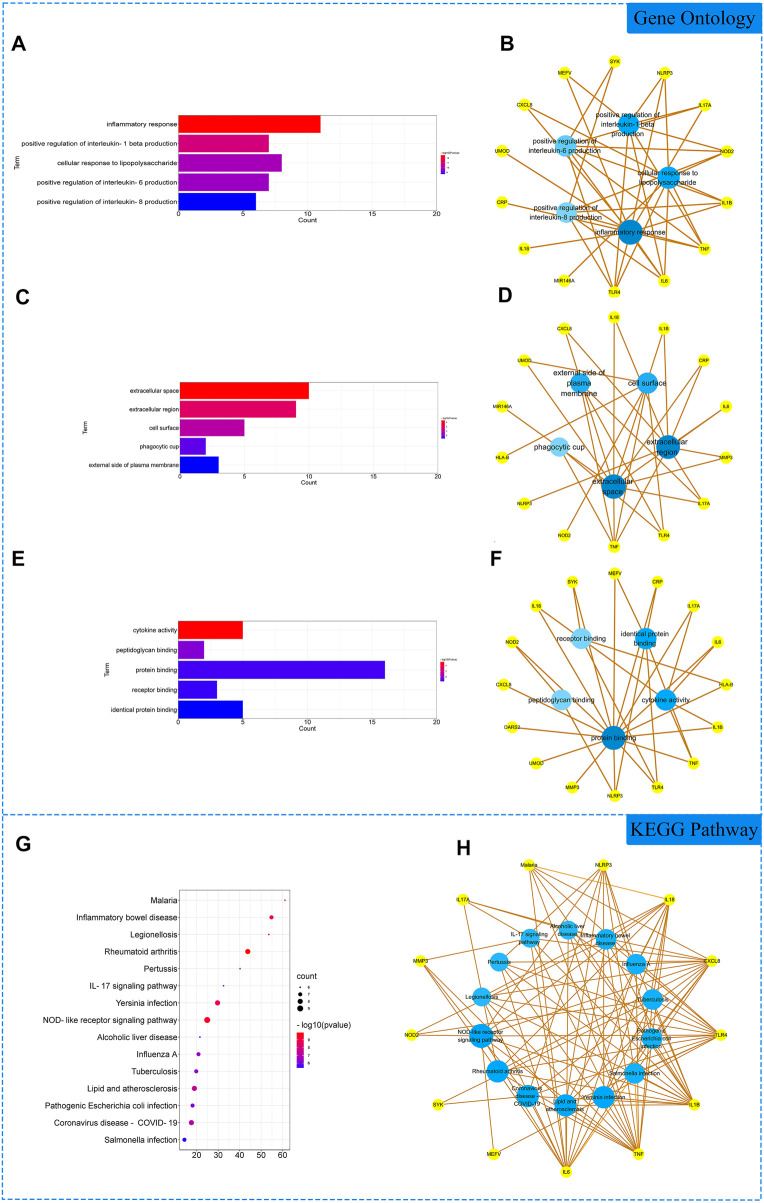
Gene Ontology (GO) and Kyoto Encyclopedia of Genes and Genomes (KEGG). Analysis of related genes. **(A)** The top 5 terms in biological processes (BP) were greatly enriched. **(B)** The subnetwork displayed the top 5 BP terms and related genes. **(C)** The top 5 terms in cellular components (CC) were greatly enriched. **(D)** The subnetwork displayed the top 5 CC terms and related genes. **(E)** The top 5 terms in molecular function (MF) were greatly enriched. **(F)** The subnetwork displayed the top 5 MF terms and related genes. **(G)** The top 15 KEGG pathways were showed. **(H)** The subnetworks displayed the top 15 KEGG pathways.

The 18 gene targets were imported into the DAVID database for enrichment analysis. Under the condition of *p* < 0.05, the GO enrichment analysis yielded 137 GO entries, including 126 BP entries, 6 CC entries, and 5 MF entries. According to the number of targets contained, the top 5 BP, CC, and MF compressions were screened. The results showed that biological processes were highly correlated with inflammation and cytokine regulation, mainly involving the inflammatory response, positive regulation of interleukin-1 beta production, and cellular response to lipopolysaccharide. Among cell components, extracellular space, extracellular region and cell surface account for a relatively large amount. In molecular functions, cytokine activity, peptidoglycan binding and protein binding were relatively high, shown in Figures 2A–F. KEGG pathway analysis yielded 48 pathways with *p* < 0.05. According to the number of targets contained, the top 15 pathways were screened. The results showed that the enriched pathways involved multiple pathways related to inflammation and immune response, mainly rheumatoid arthritis, NOD-like receptor signaling pathway, IL17 signaling pathway and other signaling pathways, shown in Figures 2G,H.

### Disease-core gene target-drug network

**FIGURE 3:**
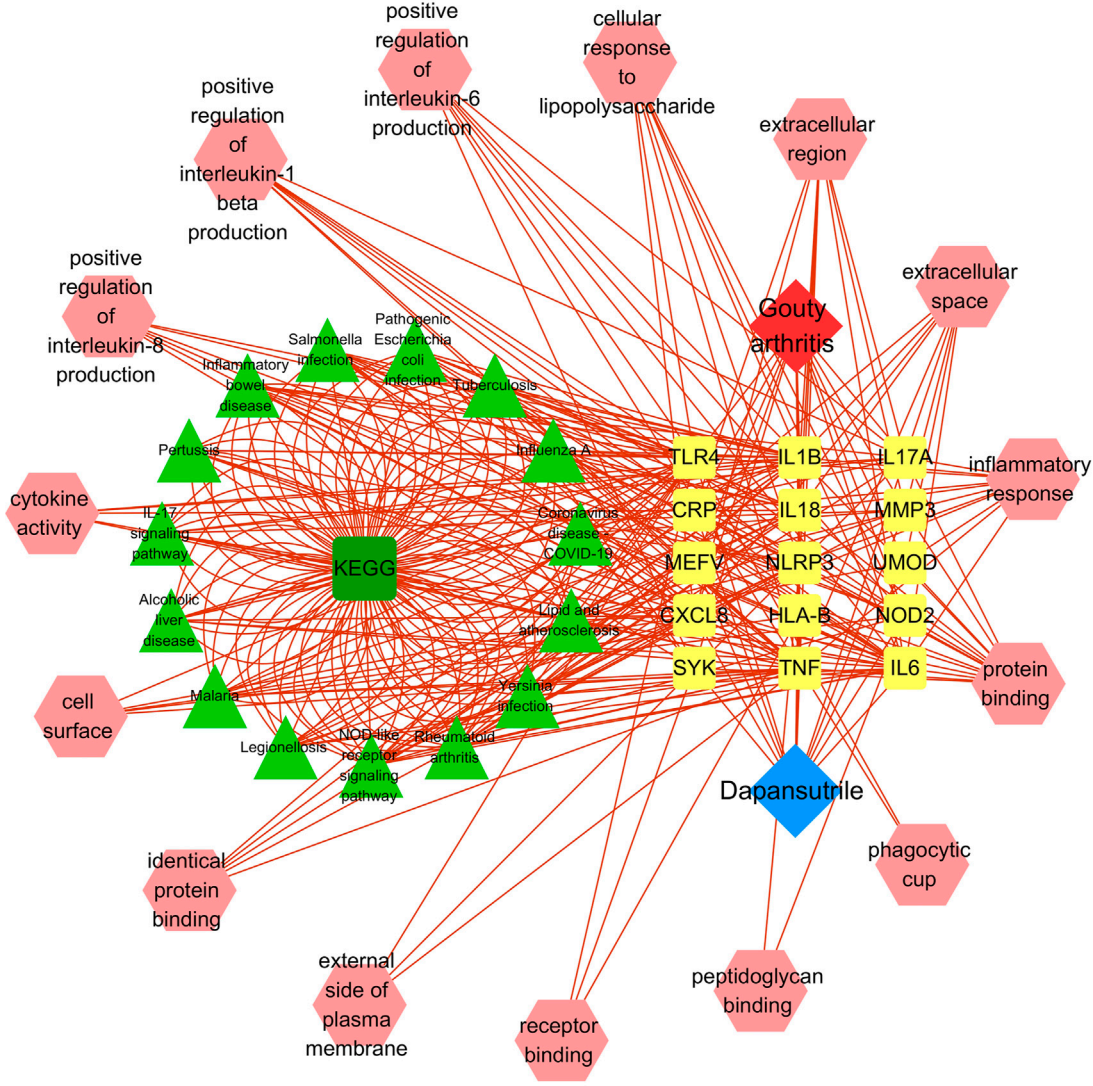
Disease-core gene target-drug network. Square nodes represent gene targets, triangular nodes represent signaling pathways (KEGG), and octagonal nodes represent gene ontology (GO) of related genes.

The disease-core gene target-drug network was constructed to show the main signal pathway and biological process of dapansutrile in the treatment of gouty arthritis, shown in Figure 3.

### Molecular docking

**FIGURE 4:**
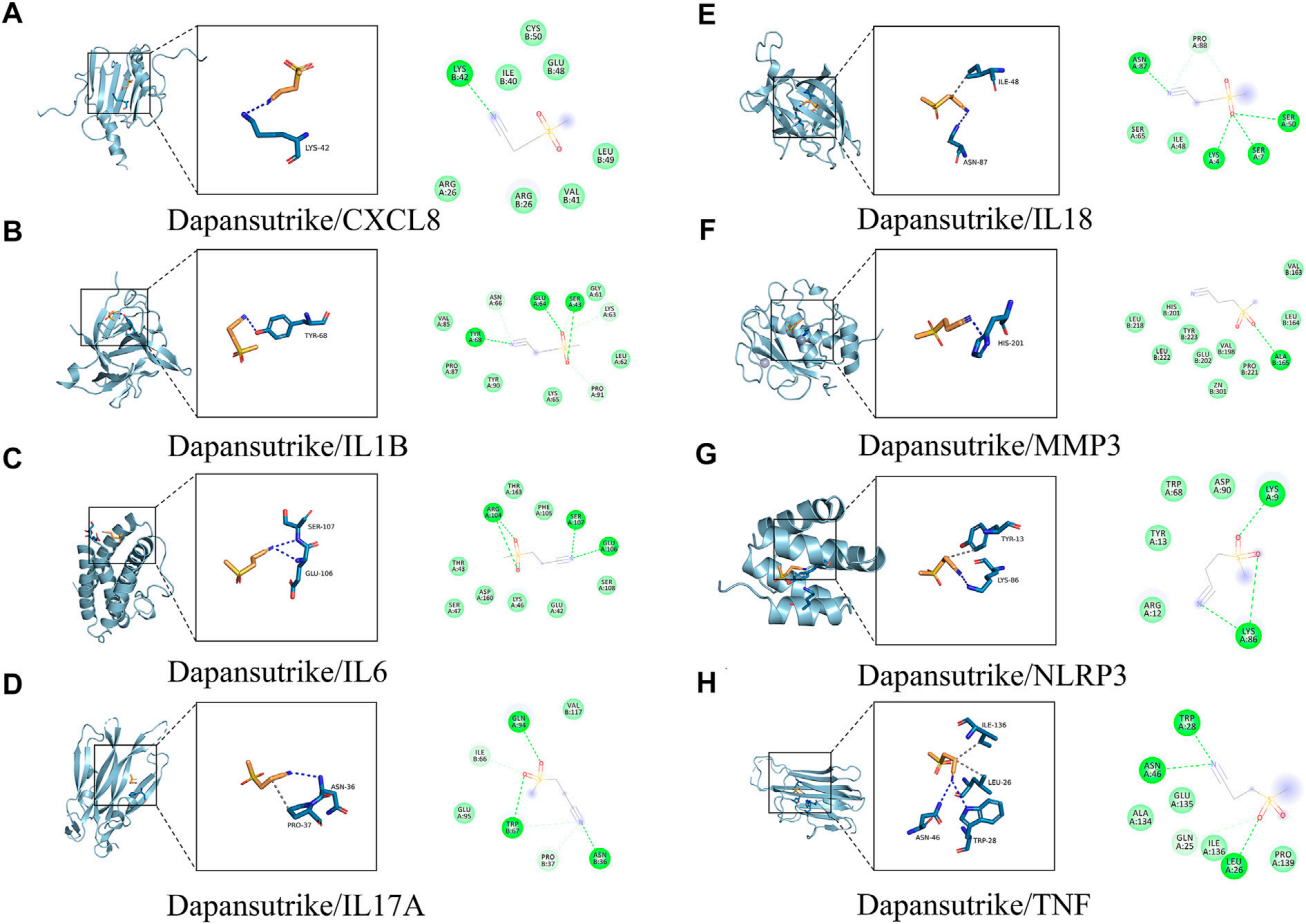
Molecular docking of active ingredients and core targets. **(A)** Dapansutrile/CXCL8, **(B)** Dapansutrile/IL1B, **(C)** Dapansutrile/IL6, **(D)** Dapansutrile/IL17A, **(E)** Dapansutrile/IL18, **(F)** Dapansutrile/MMP3, **(G)** Dapansutrile/NLRP3, **(H)** Dapansutrile/TNF.

The eight core gene targets were selected for molecular docking. The stability of receptor-ligand binding depends on the binding energy. The lower the binding energy of the complex, the more stable the receptor-ligand binding conformation. The small molecule dapansutrile interacted with various proteins mainly through hydrogen bonding and hydrophobic interactions. In addition, we were surprised that the cyano group of the small molecule dapansutrile was the main cyano group acceptor, which had hydrogen bonds with various proteins, while the sulfone group did not play the role of hydrogen bond acceptors. Moreover, we observed from IL6, IL17A, NLRP3, and TNF that the direct methylene groups of cyano and sulfone groups on dapansutrile would have hydrophobic interactions with the protein, shown in Figure 4. The results of the amino acid residues of the complexes are shown in Table 1.

**TABLE 1:** The results of the amino acid residues of the complexes.

Complex	Van Der Waals	Conventional Hydrogen Bond	Carbon Hydrogen Bond
Dapansutrile/CXCL8	CYS-50, GLU-48, ILE-40, LEU-49, VAL-41, ARG-26	LYS-42	
Dapansutrile/IL1B	VAL-85, PRO-87, TYR -90, LYS-65, LEU-62, GLY-61	TYR-68, GLU-64, SER-43	SER-43, LYS-63, ASN-66, PRO-91
Dapansutrile/IL6	THR-163, PHE-105, SER-108, GLU-42, LYS-46, ASP-160, THR-43, SER-47	ARG-104, SER-107, GLU-106	
Dapansutrile/IL17A	VAL-117, GLU-95	GLN-94, TRP-67, ASN-36	TRP-67, PRO-37, ILE-66
Dapansutrile/IL18	SER-65, ILE-48	ASN-87, SER-50, SER-7, LYS-4	PRO-88
Dapansutrile/MMP3	VAL-163, LEU-164, PRO-221, VAL-198, ZN-301, GLU-202, TYR-233, HIS-201	ALA-165	
Dapansutrile/NLRP3	ASP-90, TRP-68, TYR-13, ARG-12	LYS-9, LYS-86	
Dapansutrile/TNF	ALA-134, GLU-135, ILE-136, PRO-139	TRP-28, ASN-46, LEU-26	GLN-25

### Molecular dynamics results

**FIGURE 5:**
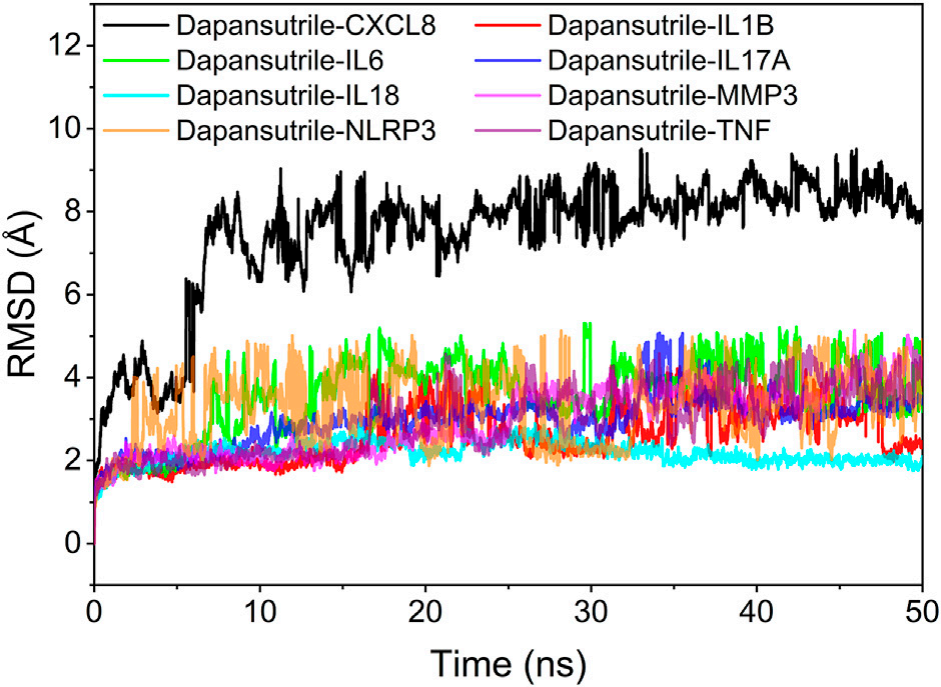
Complex root mean square deviation (RMSD) difference over time.

The root mean square skewness of molecular dynamics simulations can reflect the motion process of the complexes. The larger RMSD indicates the more intense fluctuations and motions of the complexes. The RMSD fluctuations of the eight complexes were within 5 Å for all the complexes except Dapansutrile/CXCL8 during the RMSD simulation. Therefore, based on the size and stability of RMSD, we could determine the stability of these complexes from strong to weak in order of Dapansutrile/IL18, Dapansutrile/MMP3, Dapansutrile/TNF, Dapansutrile/IL17A, Dapansutrile/IL1B, Dapansutrile/IL6, Dapansutrile/NLRP3, and Dapansutrile/CXCL8. However, RMSD results for all complexes indicated that small molecules could bind to proteins and maintain a relatively stable state. The results are shown in Figure 5.

### Combined free energy calculation results

Based on the trajectory of molecular dynamics simulation, this study calculated the binding energy by using the MM-GBSA method, which can more accurately reflect the binding mode of small molecules and target proteins. The simulation results suggested that the binding energies of Dapansutrile/CXCL8, Dapansutrile/IL1B, Dapansutrile/IL6, Dapansutrile/IL17A, Dapansutrile/IL18, Dapansutrile/MMP3, Dapansutrile/NLRP3, Dapansutrile/TNF were −2.79 ± 0.48 kcal/mol, respectively, −6.75 ± 0.52 kcal/mol, −5.67 ± 0.40 kcal/mol, −6.59 ± 0.41 kcal/mol, −3.85 ± 0.64 kcal/mol, −45.88 ± 0.85 kcal/mol, −7.06 ± 0.35 kcal/mol, −4.54 ± 0.35 kcal/mol. The value of the operation result indicates the affinity of the molecule to bind to the target protein, a lower value indicates a stronger binding affinity. The results showed that the small molecule and the corresponding proteins have strong binding affinity. The Dapansutrile/MMP3 binding was significant, the value was −45.88 ± 0.85 kcal/mol. The binding energies of these complexes are mainly contributed by van der Waals energy and electrostatic energy. The experimental results are shown in Table 2.

**TABLE 2:** Binding free energies and energy components predicted by MM/GBSA (kcal/mol).

System name	*ΔE*_vdw_	*ΔE*_elec_	*ΔG*_GB_	*ΔG*_SA_	*ΔG*_bind_
Dapansutrile/CXCL8	−10.11 ± 0.63	−10.57 ± 1.41	19.83 ± 1.54	−1.93 ± 0.11	−2.79 ± 0.48
Dapansutrile/IL1B	−17.32 ± 0.48	−19.06 ± 1.36	32.38 ± 0.96	−2.74 ± 0.03	−6.75 ± 0.52
Dapansutrile/IL6	−17.18 ± 0.68	−15.22 ± 1.44	29.73 ± 1.88	−2.98 ± 0.10	−5.67 ± 0.40
Dapansutrile/IL17A	−17.26 ± 0.49	−19.15 ± 1.20	32.50 ± 1.32	−2.68 ± 0.06	−6.59 ± 0.41
Dapansutrile/IL18	−10.72 ± 0.76	−17.01 ± 1.81	26.24 ± 1.58	−2.36 ± 0.08	−3.85 ± 0.64
Dapansutrile/MMP3	−15.44 ± 0.65	−65.59 ± 0.98	38.69 ± 0.95	−3.53 ± 0.03	−45.88 ± 0.85
Dapansutrile/NLRP3	−6.04 ± 0.70	−7.13 ± 2.28	9.31 ± 2.62	−3.19 ± 0.11	−7.06 ± 0.35
Dapansutrile/TNF	−10.89 ± 0.41	−9.73 ± 1.06	18.10 ± 1.15	−2.00 ± 0.05	−4.54 ± 0.35

*ΔE*_vdW_, van der Waals energy; *ΔE*_elec_, electrostatic energy; *ΔG*_GB_, electrostatic contribution to solvation; *ΔG*_SA_, non-polar contribution to solvation; *ΔG*_bind_, binding free energy.

### Hydrogen bond analysis

**FIGURE 6:**
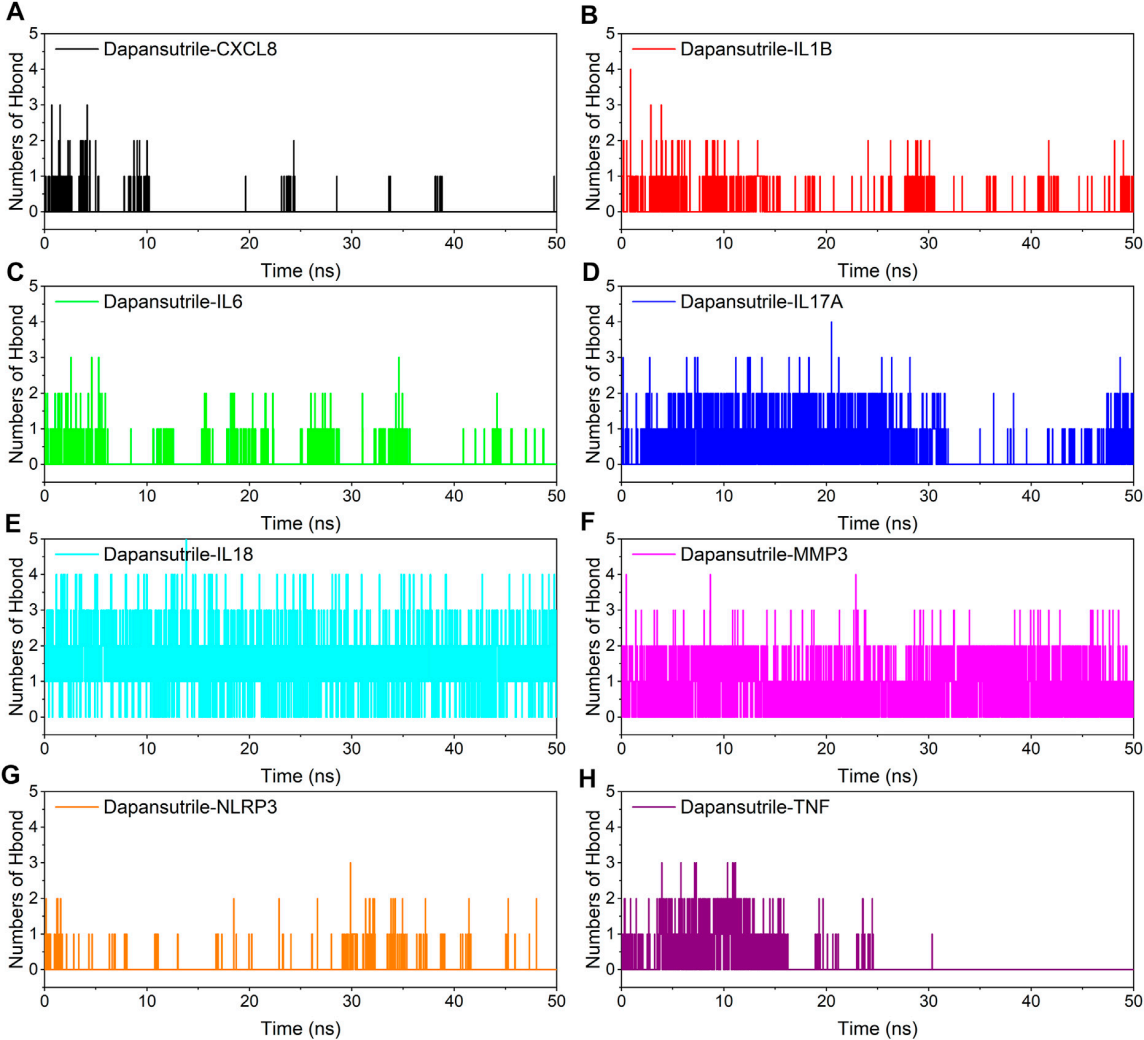
Changes in the number of hydrogen bonds between small molecule ligands and protein receptors in complex system simulations **(A)** Dapansutrile/CXCL8, **(B)** Dapansutrile/IL1B, **(C)** Dapansutrile/IL6, **(D)** Dapansutrile/IL17A, **(E)** Dapansutrile/IL18, **(F)** Dapansutrile/MMP3, **(G)** Dapansutrile/NLRP3, **(H)** Dapansutrile/TNF.

Hydrogen bonding is one of the strongest non-covalent bonding interactions, the larger the number of hydrogen bonds indicates the better binding of the complex. The experimental results showed that the optimal hydrogen bond size and density for small molecules and proteins were dapansutril/IL18 and dapansutril/MMP3, the number of hydrogen bonds was stable at about three throughout the process. This was followed closely by Dapansutrile/IL17A. The formation of hydrogen bonds in the rest of the complexes was relatively sparse. The results are shown in Figure 6.

### The stability of the target protein at the residue level

**FIGURE 7:**
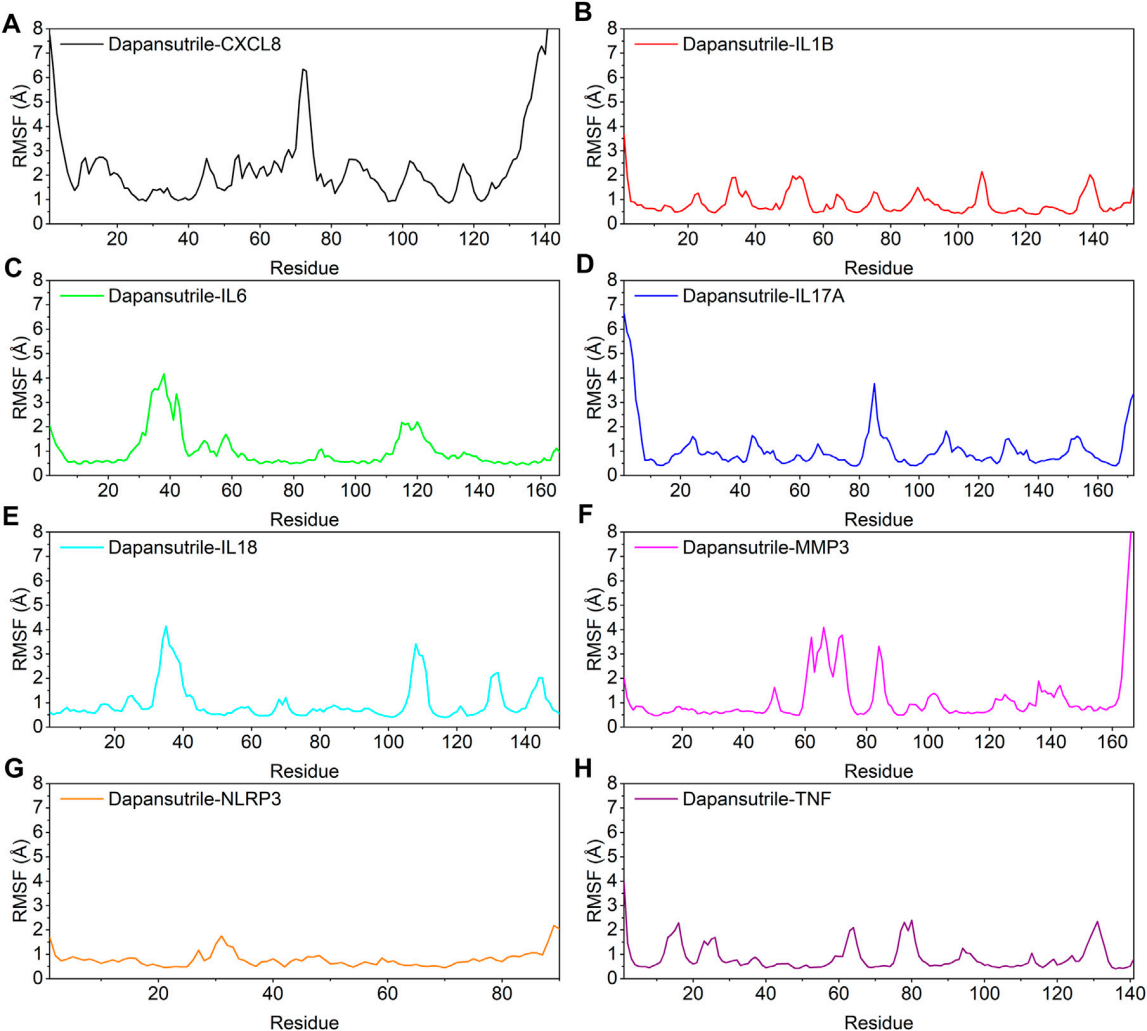
Changes in the stability of protein targets at the residue level **(A)** Dapansutrile/CXCL8, **(B)** Dapansutrile/IL1B, **(C)** Dapansutrile/IL6, **(D)** Dapansutrile/IL17A, **(E)** Dapansutrile/IL18, **(F)** Dapansutrile/MMP3, **(G)** Dapansutrile/NLRP3, **(H)** Dapansutrile/TNF.

In this study, the vibrations of each residue after binding of small molecules and proteins are explored as root mean square fluctuations (RMSF). The RMSF can reflect the flexibility of the protein during molecular dynamics simulations. The binding of small molecule drugs to proteins reduces the flexibility of the proteins, which results in stabilization of the proteins and thus their efficacy. The results showed that most of the proteins had low RMSF except for the two ends, indicating that the protein core structure has good rigidity. Notably, the overall RMSF of IL1B, NLRP3 and TNF bound to small molecules was less than 2.5 Å, indicating that these proteins are more rigid when bound to small molecules. The results are shown in Figure 7.

### Analysis of the radius of gyration

**FIGURE 8:**
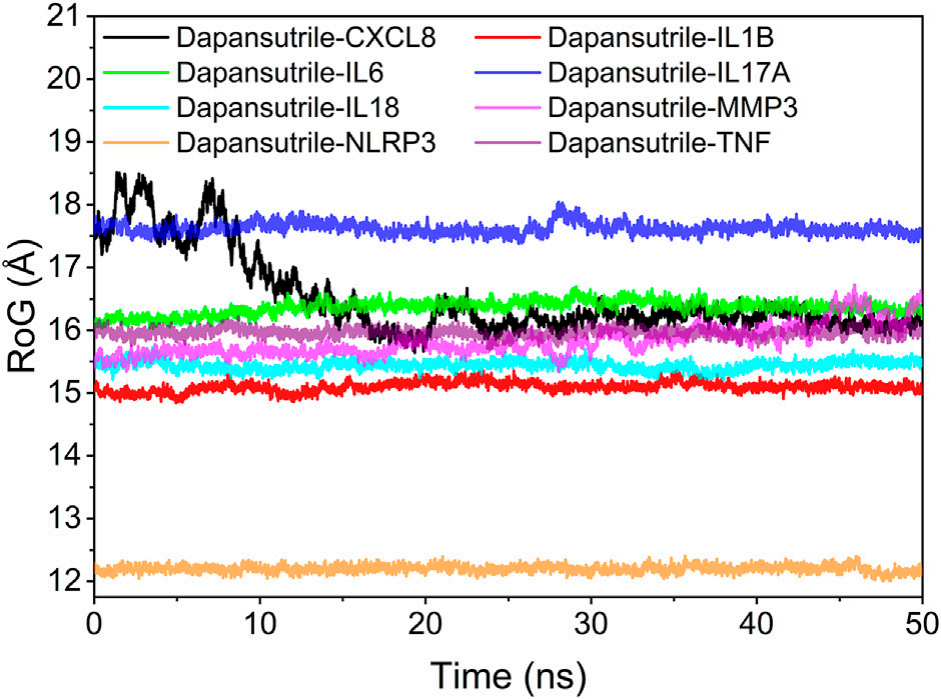
Analysis of protein folding state and overall conformation.

The radius of gyration (RoG) can reflect the degree of compactness of the complex. The results reflected the variation of RoG over time for the six complexes during the molecular dynamics simulation. The experimental results showed the degree of denseness of the complexes from largest to smallest: Dapansutrile/NLRP3, Dapansutrile/IL1B, Dapansutrile/IL18, Dapansutrile/MMP3, Dapansutrile/TNF, Dapansutrile/IL6, Dapansutrile/IL17A, and Dapansutrile/CXCL8. The simulation results indicated that dapansutrile/NLRP3, dapansutrile/IL1B, dapansutrile/IL18, and dapansutrile/MMP3 have strong binding potential. The results of RoG experiments were consistent with those of RMSD. The results are shown in Figure 8.

### Analysis of solvent accessible surface area

**FIGURE 9:**
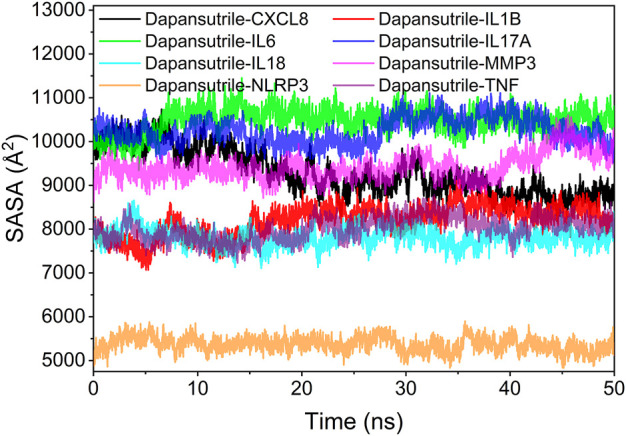
Analysis of solvent accessible surface area (SASA).

The solvent accessible surface area (SASA) indicates the area where the complex can come into contact with the aqueous solution. The contact area of the complex indicates the size of the interaction between the complex and the aqueous solution. In addition, the fluctuation of SASA responds to the exposure of the protein surface and the changes occurring in the buried area. The fluctuation analysis of SASA suggests that the fluctuations of Dapansutrile/NLRP3, Dapansutrile/TNF, Dapansutrile/IL1B, Dapansutrile/IL18, and Dapansutrile/MMP3 were small. This result implied the close interaction within the complex, which was the basis for the formation of stable binding of the complex. The results are shown in Figure 9.

## Discussion

This study explored the pharmacological mechanism of dapansutrile in the treatment of gouty arthritis by molecular docking and molecular dynamics simulation based on molecular system movement. This study found that dapansutrile may not only directly inhibit NLRP3 to reduce the inflammatory response and pyroptosis, but also hinder the chemotaxis and activation of inflammatory cells by regulating IL1B, IL6, IL17A, IL18, MMP3, CXCL8, and TNF. Firstly, dapansutrile may attenuate inflammatory responses and reduce pyroptosis by directly inhibiting the NLRP3 inflammasome and hindering the activation of downstream inflammatory inflammation. Secondly, dapansutrile may impede the activation of IL1B, CXCL8, and TNF reducing the chemotaxis and activation of inflammatory cells. Finally, dapansutrile may reduce the expression of MMP3 by regulating IL6, IL18, and IL17A, thereby degrading the extracellular matrix to treat gouty arthritis. Therefore, these results demonstrate that dapansutrile treats gouty arthritis by inhibiting the inflammatory response from multiple targets.

### Analysis of molecular docking and molecular dynamics

Molecular docking can recognize each other through the spatial matching of drug small molecule dapansutrile and protein macromolecules *in vivo*, and predict their interaction and binding mode and affinity. Therefore, the results of molecular docking can be used to explore the mechanism of action of dapansutrile on gouty arthritis.

Firstly, molecular docking experiments indicated that dapansutrile had strong affinity for the protein targets NLRP3 and MMP3. The binding of dapansutrile in IL1B, CXCL8 and TNF were relatively stable, molecular docking showed that the binding of Dapansutrile/IL1B and Dapansutrile/IL18 is mainly maintained by hydrogen bonding and hydrophobic interaction. Dapansutrile/CXCL8 and Dapansutrile/MMP3 were mainly through hydrophobic interaction. Dapansutrile combined with IL6, IL18, and IL17A can form stable complexes, but there were some abnormal fluctuations, which may be due to the influence of the number and angle of binding bonds.

Secondly, based on the trajectory of the molecular dynamics simulation, we calculated the binding energy using the MMGBSA method, which could more accurately reflect the binding mode of small molecules and target proteins. In the molecular dynamics simulation, the RMSDs of Dapansutrile/NLRP3 converged gradually in the first 5 ns of the simulation and preserved stable fluctuations in subsequent simulation. The RMSF results suggested that the overall RMSF of IL1B and TNF was less than 2.5 Å when they were bound to small molecules. These proteins are more rigid when bound to small molecules. The radius of gyration suggested that Dapansutrile/IL18 and Dapansutrile/MMP3 have stable fluctuations in size, which means they have high binding potential. Hydrogen bonding is also an important basis for the formation of stable binding between small molecule drugs and protein targets. The results of this study showed that the number of hydrogen bonds in Dapansutrile/IL18 and Dapansutrile/MMP3 were the best in terms of size and density. The number of their hydrogen bonds was stable throughout at about three. And all eight complexes can form stable hydrogen bonds. Therefore, the results of hydrogen bonding analysis showed that all the eight complexes had the basis for forming stable bonds.

Finally, the binding free energy consists mainly of non-bonding interactions (such as: Van der Waals interactions, electrostatic interactions and hydrogen bonding interactions). Because only non-bonding interactions are generally present in the actual drug small molecule (ligand) and protein (receptor) complex systems. The drug molecule binds reversibly to the proteins through non-bonding interactions, and this binding is more favorable for its own metabolism and excretion. Therefore, the results of free energy of binding were the comprehensive evaluation of the binding stability of drug and protein targets in this study. We analyzed our binding free energy consequences and other experimental results to derive a comprehensive ranking of the complex binding stability from strong to weak Dapansutrile/MMP3, Dapansutrile/NLRP3, Dapansutrile/IL1B, Dapansutrile/IL17A, Dapansutrile/IL6, Dapansutrile/TNF, Dapansutrile/IL18, Dapansutrile/CXCL8.

### Dapansutrile may attenuate the inflammatory response in the treatment of gouty arthritis by inhibiting the NACHT, LRR, and PYD domains-containing protein 3 inflammasome

Dapansutrile may inhibit the activation of downstream inflammatory signaling pathways and reduce cell death by directly inhibiting the NLRP3 inflammasome.

Bioinformatics analysis suggested that dapansutrile can attenuate inflammatory responses and reduce pyroptosis by directly inhibiting the NLRP3 inflammasome and hindering the activation of downstream inflammatory inflammation. As a key component of inflammatory activation, NLRP3 plays a crucial role in innate immunity and inflammation. NLRP3 has a regulatory role in inflammation, immune response and cellular scorching as an upstream activator of NF-kappaB signaling. Analysis of protein interaction network PPI suggested that NLRP3 was closely related to inflammatory responses targets. KEGG signaling pathway analysis showed that NLRP3 was involved in protein metabolism and NOD-like receptor signaling pathway. GO analysis suggested that NLRP3 was involved in peptidoglycan binding.

The NLRP3 inflammasome is a complex containing the NLRP3 protein, the adaptor protein apoptosis-associated speck-like protein (ASC) and procaspase-1 (Inoue and Shinohara, 2013a, 2013b; Abderrazak et al., 2015; Song and Li, 2018). The interaction among the three proteins tightly regulates the function of the inflammasome to ensure immune activity only when appropriate (Shao et al., 2015). NLRP3 can trigger caspase-1 self-activation (Schroder and Tschopp, 2010). In the presence of immune activators [such as: Pathogen-associated molecular pattern molecules (PAMPs), danger-associated molecular patterns (DAMPs), other exogenous invaders, or environmental stress], NLRP3 opens and allows interaction between NLRP3 and the pyrin domain (PYD) in ASC. Subsequently, the caspase recruitment domain (CARD) of ASC in turn recruits the CARD domain on procaspase-1 for binding, resulting in the generation of the NLRP3 inflammasome (Martinon et al., 2006; Willingham et al., 2009; Schroder and Tschopp, 2010; Shao et al., 2015), NLRP3 inflammasome formation also triggers pyroptosis (Jorgensen and Miao, 2015). Gouty arthritis is driven by macrophage uptake of deposited sodium urate crystals and subsequent activation of the NLRP3 inflammasome (Martinon et al., 2006; Cavalcanti et al., 2016). However, dapansutrile is an orally active β-sulfonitrile molecule that inhibits NLRP3 inflammasome activation (Marchetti et al., 2018a; Sánchez-Fernández et al., 2019; Wohlford et al., 2020). Research showed that the concentrations of dapansutrile were found to inhibit NLRP3-ASC and NLRP3-caspase-1 interaction *in vitro* at 1 μM or less. Interestingly, in LPS-stimulated human blood-derived macrophages, dapansutrile reduced IL1B levels by 60% and IL18 by 70%, *in vitro* at concentrations 100-fold lower than plasma concentrations safely achieved in humans (Marchetti et al., 2018a; Wohlford et al., 2020). Alba Sánchez-Fernández et al. (2019) found that prophylactic oral administration of dapansutrile resulted in a significant (2- to 3-fold) reduction in the protein levels of IL1B and IL18 as well as IL6 and TNFα in the spinal cord of EAE mice.

Bertinaria discussed whether the bond between dapansutrile and NLRP3 was covalent or non-covalent (Bertinaria et al., 2018). In fact, small molecules can have multiple binding sites with protein ligands at the same time. In our simulation studies, we found that the cyano and sulfone groups of dapansutrile could form hydrogen bonds with the protein NLRP3. Moreover, the drug usually binds to the protein target and acts through non-covalent bonds. Therefore, it is more reasonable to conclude that dapansutrile inhibits NLRP3 by forming a stable bond with NLRP3 through non-covalent bonds. At the same time, our results showed that dapansutrile may not only act on NLRP3, but also block the downstream signaling pathway of NLRP3 by IL1B, IL18, IL6, thus reducing the inflammatory response. It is possible that our simulation results can better explain the strong NLRP3 inhibitory and anti-inflammatory effects of dapansutrile.

Therefore, dapansutrile may attenuate inflammation and reduces pyroptosis by inhibiting the NLRP3 inflammasome to treat gouty arthritis.

### Dapansutrile in the treatment of gouty arthritis by inhibiting the occurrence of inflammation and chemotaxis

Dapansutrile may reduce the inflammatory response and the chemotaxis of inflammatory cells by blocking the activation of IL1B, CXCL8, and TNF for the treatment of gouty arthritis.

Dapansutrile may impede the activation of IL1B, CXCL8, and TNF reducing the chemotaxis and activation of inflammatory cells. IL1B is an important mediator of inflammatory response, and it is involved in various cellular activities such as cell proliferation, differentiation and apoptosis. IL1B is also involved in the pathogenesis of osteoarthritis. KEGG signaling pathway analysis suggested that IL1B regulated rheumatoid arthritis and glucocorticoids. GO analysis indicated that IL1B was associated with protein domain-specific binding. CXCL8 (also known as IL8) is a chemokine that attracts neutrophils, basophils and T cells. It is not only involved in neutrophil activation and chemotaxis, but also has a role in systemic inflammatory response syndrome (SIRS). KEGG signaling pathway analysis included cellular senescence and MIF-mediated glucocorticoid regulation. GO analysis included chemokine activity and interleukin 8 receptor binding. Protein interaction network analysis indicated that IL1B, CXCL8, and TNF were jointly engaged in the chemotaxis and activation of inflammatory cells. TNF is a multifunctional pro-inflammatory cytokine. This cytokine is mainly secreted by macrophages, it causes fever by direct action or through IL1B secretion, and has been implicated in the induction of cachexia. KEGG signaling pathway analysis suggested that TNF regulated inflammatory response and inflammatory bowel disease. GO analysis indicated that TNF affected cytokine activity.

Tengesdal et al. (2021) found that dapansutrile reduced pSTAT3 (Y705) by 82% and IL6 expression by 53%. IL6 binds to its receptor complex in IL6R/gp130 to activate downstream Janus kinases (JAKs), which subsequently activate signal converters and activators of transcription 3 (STAT3) through phosphorylation of tyrosine 705 (Mauer et al., 2015). König et al. (2021) found that activation of TNF was dependent on IL6 signaling, and TNF also limited the action of IL1B. Furthermore, activation of IL6 transsignal must be “downstream” of TNF signaling (König et al., 2021). Meanwhile, IL1B is highly expressed NF-κB activator in triple negative breast cancer (TNBC) (Ignacio et al., 2019). NF-κB can increase the expression of TNF-α and IL6 (Huang et al., 2008; Liu et al., 2013). Therefore, IL1B and TNF-α can regulate each other. And You et al. (2021) demonstrated that IL1B enhanced the expression level of CXCL8 in TNBC cells.

Therefore, dapansutrile may reduce the chemotaxis and activation of inflammatory cells for the treatment of gouty arthritis by blocking the activation of IL1B, CXCL8, and TNF.

### Dapansutrile in the treatment of gouty arthritis by degrading extracellular matrix

Dapansutrile may regulate extracellular matrix degradation by reducing MMP3 expression through IL6, IL18, and IL17A in the treatment of gouty arthritis.

Dapansutrile may reduce the expression of MMP3 by regulating IL6, IL18, and IL17A, thereby degrading the extracellular matrix to deal with gouty arthritis. IL6 is mainly produced at sites of acute and chronic inflammation, and it has been implicated in various inflammation-related disease states, including diabetes and systemic rheumatoid arthritis. Protein interaction network analysis showed that IL6, IL18, IL17, and MMP3 were closely connected with each other. KEGG signaling pathway analysis showed that IL6 was involved in dendritic cell developmental lineage pathways and cellular senescence. GO analysis included signaling receptor binding. IL17A is involved in inducing the production of inflammatory molecules, chemokines, antimicrobial peptides and remodeling proteins. IL17A plays a key role in inducing innate immune defenses, and IL17A stimulates non-hematopoietic cells and promotes the production of chemokines, which attract bone marrow cells to sites of inflammation. KEGG signaling pathway analysis included MIF-mediated glucocorticoid regulation and IL17 family signaling pathways. GO analysis included cytokine activity. IL18 is associated with tissue and organ damage and plays an important role in potentially fatal diseases characterized by cytokine storm. KEGG signaling pathway included MIF-mediated glucocorticoid regulation and IL-1 family signaling pathway. MMP3 is involved in the breakdown of extracellular matrix in arthritis and metastasis. MMP3 is regarded as involved in wound repair, atherosclerosis progression and tumor initiation. KEGG analysis included Gastrin-CREB signaling through PKC and MAPK. GO analysis included calcium binding and metallopeptidase activity.

Studies have shown that dapansutrile can inhibit the IL6/STAT3 axis to inhibit breast cancer metastasis (Siersbæk et al., 2020; Tengesdal et al., 2021). Tantilertanant et al. (2019) found that circulating tension-upregulated IL6 increased MMP3 expression in human periodontal ligament cells. The IL6 amplifier (IL6 Amp) is an amplification mechanism, the synergistic interaction of STAT3 with nuclear factor-κB (NF-κB) produces IL6 and various other cytokines and chemokines (Hirano, 2010; Murakami et al., 2013; Atsumi et al., 2014). Moreover, study found that IL6 could activate NF-κB through the IL6-STAT3 axis (Hirano, 2021). Wang et al. (2019) found that IL18 promoted MMP secretion in human periodontal ligament fibroblasts by activating NF-κB signaling. Koenders et al. (2005) found that IL17A promoted gastric cancer invasiveness through NF-κB-mediated expression of MMP2 and MMP9. Therefore, IL17A and IL18 regulate MMP3 expression through NF-κB. At the same time, the proinflammatory cytokines IL1B and TNF-α produced after activation of the NLRP3 inflammasome can also promote the expression of MMP3 (Burrage et al., 2006; You et al., 2021).

Therefore, Dapansutrile may decrease the expression of MMP3 by regulating IL6, IL18, and IL17A, thereby degrading the extracellular matrix for the treatment of gouty arthritis.

### Analyze the value of dapansutrile in the treatment of gouty arthritis

Gouty arthritis is a metabolic rheumatic disease caused by disorders of purine metabolism and reduced synthesis or excretion of uric acid. Therefore, the treatment of gouty arthritis mainly revolves around correcting the metabolic abnormalities and reducing the inflammatory response of the body. Current studies have shown that dapansutrile alleviates the clinical symptoms of gouty arthritis by suppressing the inflammatory response, and that dapansutrile has good therapeutic effects and few side effects.

The results of our study further validated the important role of dapansutrile in reducing the inflammatory response. And we believed that dapansutrile could reduce the inflammatory response and body damage not only through NLRP3 but also through other protein targets (such as: MMP3, IL1B, and IL18). Moreover, the further development of related drugs should focus on and learn from some of the characteristics of dapansutrile. Firstly, dapansutrile has a very simple structure, and its molecular weight is around one hundred. The simple molecular structure allows dapansutrile to have satisfactory transmembrane ability, the excellent transmembrane ability provides the basis for dapansutrile to inhibit the intracellular inflammatory response. Secondly, dapansutrile has good absorption, which can effectively improve drug utilization and reduce drug dosage, thus reducing drug side effects. Based on the good absorption properties, dapansutrile is currently being developed for oral administration. Finally, the clear and reliable biosafety proof is the biggest advantage of dapansutrile. The development and improvement of any related drug must be based on the principle that there is no or minimal biological toxicity.

The summary of the mechanisms analysis of dapansutrile in the treatment of gouty arthritis is shown in Graphical Abstract.

## Conclusion

This study explored the pharmacological mechanism of dapansutrile in the treatment of gouty arthritis by molecular docking and molecular dynamics simulation based on molecular system movement. This study found that Dapansutrile may not only directly inhibit NLRP3 to reduce the inflammatory response and pyroptosis, but also hinder the chemotaxis and activation of inflammatory cells by regulating IL1B, IL6, IL17A, IL18, MMP3, CXCL8, and TNF.

Therefore, Dapansutrile treats gouty arthritis by attenuating inflammatory response, inflammatory cell chemotaxis and extracellular matrix degradation by acting on multiple targets.

## Data Availability

The original contributions presented in the study are included in the article/Supplementary Materials, further inquiries can be directed to the corresponding authors.

## Glossary

ACR: The American College of Rheumatology
AGA: Acute gouty arthritis
ASC: Apoptosis-associated speck-like protein
BP: Biological process
CARD: The caspase recruitment domain
CC: Cellular component
CXCL8: C-X-C motif ligand 8
DAMPs: Damage-associated molecular patterns
GO: Gene ontology
HF: Hartree-Fock
IL17A: Interleukin 17A
IL18: Interleukin 18
IL1B: Interleukin 1 beta
IL6: Interleukin 6
KEGG: Kyoto Encyclopedia of Genes and Genomes
LRR: Leucine-rich repeat
MAPK: Mitogen-activated protein kinases
MD: Molecular dynamics
MF: Molecular function
MIF: Macrophage migration inhibitory factor
MMP2: Matrix metalloproteinase 2
MMP3: Matrix metalloproteinase 3
MMP9: Matrix metalloproteinase 9
MM-PBSA: Molecular Mechanics-Poisson Bolzmann Surface Area
MSU: Monosodium urate
NACHT: Nucleotide-binding and oligomerization
NF-κB: Nuclear Factor Kappa B
NLRP3: The NACHT, LRR, and PYD domains-containing protein 3
NLRs: NOD-like receptors
NSAIDs: Non-steroidal anti-inflammatory drugs
NYHA: New York Heart Association
PAMPs: Pathogen-associated molecular pattern molecules
PKC: Protein Kinase C
PME: The Particle-Mesh-Ewald
PPI: Protein-protein interaction
PRRs: Pattern recognition receptors
PYD: Pyrin domain
RMS: Root mean square
RMSD: Root mean square deviation
RMSF: Root mean square fluctuation
SASA: Solvent accessible surface area
SIRS: Systemic inflammatory response syndrome
STAT3: Signal transducer and activator of transcription 3
TNBC: Triple negative breast cancer
TNF: Tumour necrosis factor alpha
Vdw: Van der Waals force
